# Acute Toxicity Assessment: Macroscopic and Ultrastructural Effects in Mice Treated with Oral Tetrodotoxin

**DOI:** 10.3390/toxins11060305

**Published:** 2019-05-29

**Authors:** Paula Abal, M. Carmen Louzao, Natalia Vilariño, Mercedes R. Vieytes, Luis M. Botana

**Affiliations:** 1 Departamento de Farmacología, Facultad de Veterinaria, Universidad de Santiago de Compostela, 27002 Lugo, Spain; paula.abal@usc.es (P.A.); natalia.vilarino@usc.es (N.V.); 2 Departamento de Fisiología, Facultad de Veterinaria, Universidad de Santiago de Compostela, 27002 Lugo, Spain; mmercedes.rodriguez@usc.es

**Keywords:** tetrodotoxin, macroscopic alterations, ultrastructural changes, gastrointestinal effect, mitochondria swelling, rough endoplasmic reticulum swelling

## Abstract

Tetrodotoxin (TTX) is an extremely toxic marine compound produced by different genera of bacteria that can reach humans through ingestion mainly of pufferfish but also of other contaminated fish species, marine gastropods or bivalves. TTX blocks voltage-gated sodium channels inhibiting neurotransmission, which in severe cases triggers cardiorespiratory failure. Although TTX has been responsible for many human intoxications limited toxicological data are available. The recent expansion of TTX from Asian to European waters and diversification of TTX-bearing organisms entail an emerging risk of food poisoning. This study is focused on the acute toxicity assessment of TTX administered to mice by oral gavage following macroscopic and microscopic studies. Necropsy revealed that TTX induced stomach swelling 2 h after administration, even though no ultrastructural alterations were further detected. However, transmission electron microscopy images showed an increase of lipid droplets in hepatocytes, swollen mitochondria in spleens, and alterations of rough endoplasmic reticulum in intestines as hallmarks of the cellular damage. These findings suggested that gastrointestinal effects should be considered when evaluating human TTX poisoning.

## 1. Introduction

Tetrodotoxin (TTX) is a naturally occurring marine neurotoxin whose production is attributed to several phyla of bacteria [1,2]. Microbial producers of TTX can directly excrete the toxin into the aquatic environment. Compound transfer can also occur from bacteria to host animals that include gastropods, crabs, starfish, octopus, frogs, or marine and terrestrial flatworms [3,4]. The most common route of human tetrodotoxin poisoning is consumption of the puffer fish (family *Tetraodontidae*), to which TTX owes its name [5]. Symptoms of TTX poisoning are neuromuscular, gastrointestinal, cardiovascular and respiratory [6]. TTX sometimes is lethal, being responsible for the highest mortality rate of all marine intoxications [7]. This toxin has the potential to pose a food safety risk to humans, even though it is not yet tested routinely at the international level [8]. 

Until 2007, TTX has been associated predominantly with Asian countries, nevertheless the presence of the toxin was recently reported in European molluscs from the Atlantic coast [9,10] and the Mediterranean Sea [11]. This geographic expansion may be due to lessepsian migration of toxin-bearing animals through the Suez Canal [12] along with increasing water temperatures worldwide [13]. In Europe, only the main fish species containing TTXs (*Tetrodontidae*, *Molidae*, *Diodontidae* and *Cnthigasteridae*) are not allowed to be placed on the market [14]. However, the recent discovery of TTX-contaminated shellfish such as oysters and mussels in European waters [9,11,15] has prompted the consideration of TTX as an emerging threat for food safety in the EU. 

Although TTX has been known to cause many human intoxications, toxicity data are scarce [16]. TTX exhibits its toxicity by targeting voltage-gated sodium channels (Na_v_) [17,18]. TTX occludes the pore thus suppressing the influx of sodium ions into the cell [19]. This blockage abolishes the propagation of action potentials in cell membranes, thereby paralyzing nerve and muscle function [20]. The clinical signs of TTX poisoning are: tongue and lips tingling, perioral paresthesia, numbness of extremities, paralysis, and muscular incoordination. The action of TTX on sodium channels and the limited ability of TTX to cross the blood–brain barrier opens the door to its potential application in neuroscience and medicine as an anesthetic and analgesic drug [21,22,23,24]. Severe intoxication may result in death due to respiratory muscle paralysis and cardiovascular arrest that can occur as early as 17 minutes after ingestion [25]. This short period of time suggest a rapid absorption of TTX in the digestive tract, although its presence lasts for days after exposure in humans as it is shown by the analysis of blood [26]. In the absence of human data, the best option to determine TTX toxicity is to use rodents. Pharmacokinetic studies in rats and mice confirmed this fast absorption since the toxin was detected in blood minutes after TTX administration [27,28]. *In vivo* studies in mice described the lethal dose 50 (LD_50_) by intraperitoneal (i.p.) injection as 8.2–10.7 µg TTX/kg bw by subcutaneous (s.c.) as 10–12.5 µg TTX/kg bw [29]. A recent acute oral study with mice established that the LD_50_ is 232 µg TTX/kg bw and that 25 µg/kg bw had non observed adverse effects [27]. Based on this value the European Food Safety Authority (EFSA) Panel on Contaminants in the Food Chain (CONTAM) derived the acute reference dose (ARfD) of 0.25 µg/kg applying to TTX and its analogues [29]. Recently, TTX was included in a Dutch seafood monitoring program which established the EFSA proposed limit of 44 µg TTX/kg shellfish [15,29]. In order to evaluate the seafood safety risk to consumers more toxicological studies are needed. 

The goal of the present study was to contribute to the knowledge on the oral acute toxic effects of TTX in mice with particular focus on the macroscopic and ultrastructural alterations. 

## 2. Results

### 2.1. Animal Symptoms and Mortality

In this acute toxicity study, animals dosed with 125 µg/kg bw TTX or lower (25–75 µg/kg bw) survived the entire experiment. However, mice exposed to 125 µg/kg bw were apathetic, some of them showed symptoms of discomfort. The dose of 250 µg/kg bw TTX induced apathy and caused 57% death. At the dose of 500 µg/kg bw, TTX-treated animals suffered signs of toxicity such as paralysis of extremities and seizures and mortality increased up to 80%. At the highest dose of 1,000 µg/kg bw TTX clinical signs were similar to the previous dose but with higher incidence (Table 1).

### 2.2. Necropsy

**Figure 1:**
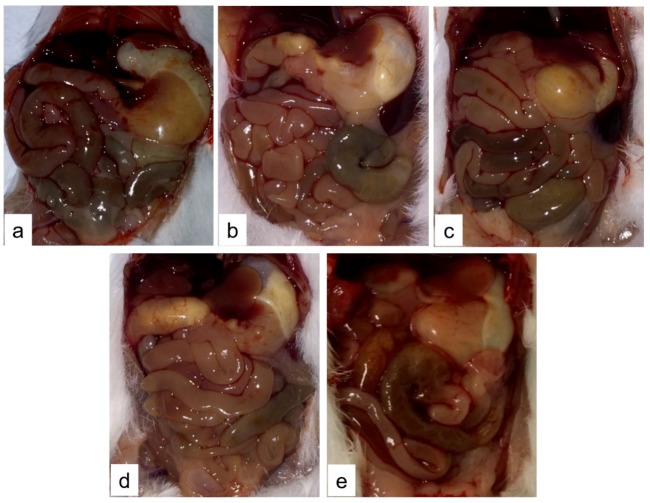
Representative image of organs of control mice (**a**) or mice treated with 125 (**b**), 250 (**c**), 500 (**d**), and 1000 µg/kg TTX bw (**e**).

**Figure 2:**
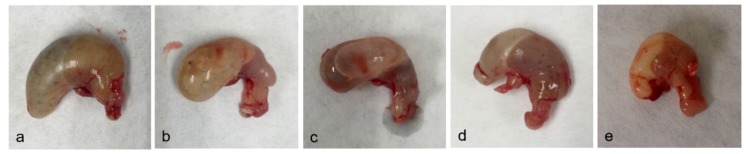
Representative image of stomachs of control mice (**a**) or mice treated with 125 (**b**), 250 (**c**), 500 (**d**), and 1,000 µg/kg TTX bw (**e**).

After dying or at the scheduled euthanasia (2 h after TTX administration) necropsy was performed on control and TTX-treated mice. Macroscopically, animals administered with 125, 250, and 500 µg/kg bw TTX showed swollen abdomen with liquid content. Mice treated with those doses presented alterations in small intestine (Figure 1) and stomach (Figure 2). Briefly, in control mice small intestine was full of digested content (Figure 1a) also stomach was full of food (Figure 2a). Small intestine of all treated mice showed lower content than in control animals but with some accumulation of fluid (Figure 1c,d), while mice treated with 1,000 µg TTX/kg bw had almost no fluid in its lumen (Figure 1e). Animals treated with TTX showed liquid and gas accumulation in the stomach that was evident at low-medium doses (125–500 µg/kg bw) (Figure 2b–d). 

**Figure 3:**
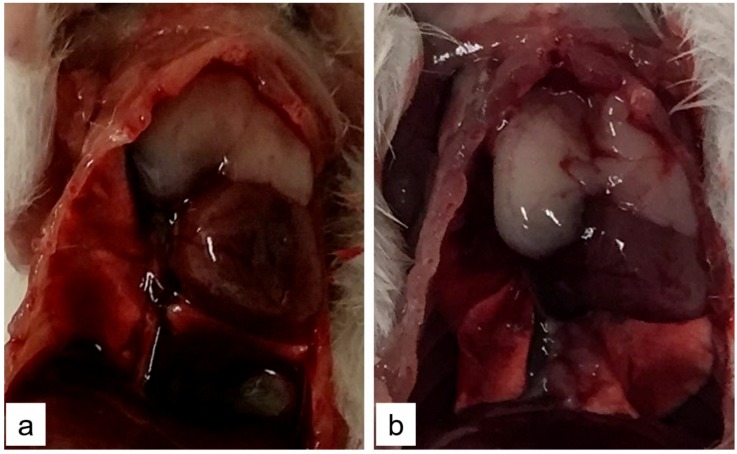
Representative image of heart of control mice (**a**) or mice treated with 1000 µg/kg bw TTX (**b**). The reduction in size of the heart reflected the stiffness of the organ and the absence of intracardiac blood.

TTX-treated mice did not present any evidence of lung damage however hearts of mice treated with the highest dose (1000 µg/kg bw of TTX) were stiff and without blood content (Figure 3). Other mice organs showed no macroscopic modifications. 

### 2.3. Ultrastructural Examination

**Figure 4:**
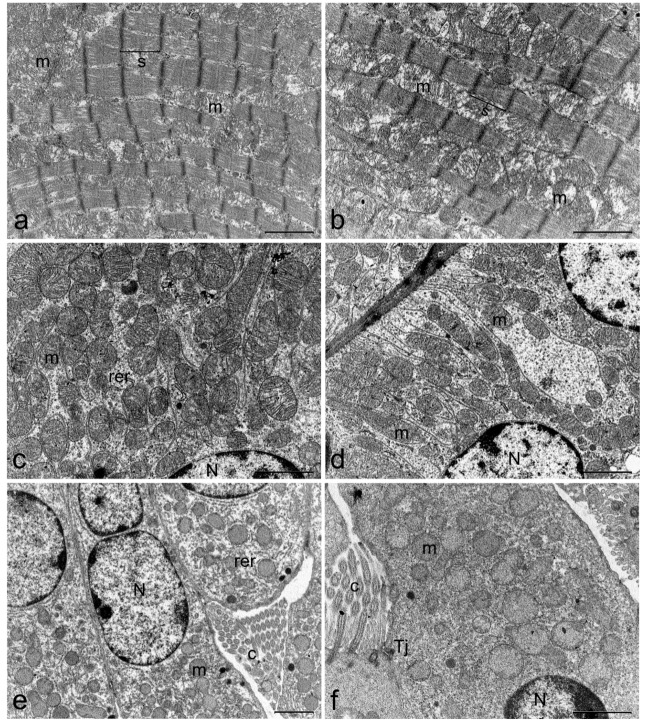
Representative electron micrograph showing ultrastructure of heart (**a**,**b**), kidney (**c**,**d**), and lung (**e**,**f**) of control mice (first column) or mice treated with 500 µg/kg bw TTX (second column). (**a**) scale bar = 1 µm, (**b**–**f**) scale bar = 2 µm. Cilia (c), mitochondria (m), nucleus (N), rough endoplasmic reticulum (rer), sarcomere (s), tight junction (Tj).

**Figure 5:**
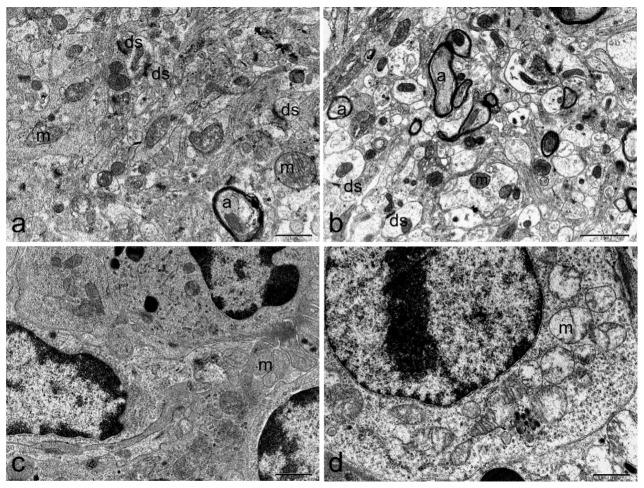
Representative electron micrograph showing ultrastructure of brain (**a**,**b**) and spleen (**c**,**d**) of control mice (first column) or mice treated with 500 µg/kg bw TTX (second column). Swollen mitochondria were detected in spleen cells. (**a**,**c**,**d)** scale bar = 1 µm, (**b**) scale bar = 2 µm. Axon (a), dendritic synapse (ds), mitochondria (m).

**Figure 6:**
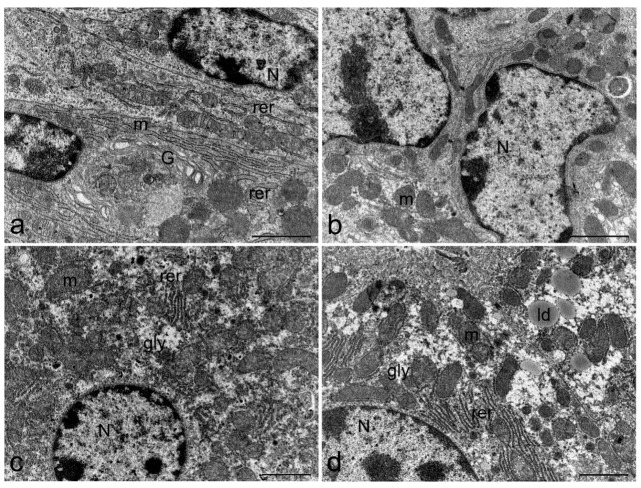
Representative electron micrograph showing ultrastructure of stomach (**a**,**b**) and liver (**c**,**d**) from control mice (**a**,**c**) and mice treated with 500 µg/kg bw TTX (**b**,**d**). Increased presence of lipid droplets was found in hepatocytes of TTX-treated mice. Scale bar = 2 µm, glycogen (gly), lipid droplets (lp), mitochondria (m), nucleus (N), and rough endoplasmic reticulum (rer).

**Figure 7:**
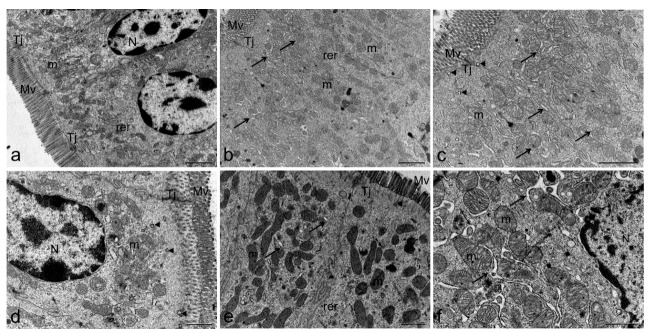
Representative electron micrograph showing ultrastructure of the small (**a**–**c**) and large intestine (**d**–**f**) from control mice (**a**,**d**) and mice treated with 500 µg/kg bw TTX (**b**,**c**,**e**,**f**). Dilation of rer was detected in small and large intestine. (**a**–**c**) scale bar = 2 µm, (**d**–**f**) scale bar = 1 µm. endosome (arrow head), microvilli (Mv), mitochondria (m), nucleus (N), rough endoplasmic reticulum (rer), swollen rer (arrow), and tight juntion (Tj).

At the end of treatment, organs including hearth, liver, kidney, brain, spleen, stomach, small and large intestine or lungs were extracted and subjected to examination. Transmission electron microscopy (TEM) studies showed no alterations at heart, kidney, lung, and brain level. Ultrastructural modifications were identified in small and large intestine, spleen and liver of TTX treated mice. Representative microphotographs of organs from a mouse of the 500 µg/kg bw dose group were presented in Figure 4, Figure 5, Figure 6 and Figure 7. TEM analysis of the cardiac tissue from TTX-treated mice revealed mitochondria regularly packed with abundant and differentiated cristae in electron transparent matrix and sarcomeres well structured (Figure 4b). Kidney images showed tubular lining cells with numerous mitochondria and ribosomes in the cytoplasm and nucleus with lightly packed chromatin in both control and TTX-treated mice (Figure 4c,d). Lung cells (Figure 4e,f) showed organelles, cilia and tight junctions well preserved. In brain cells (Figure 5a,b), synaptic unions correctly conformed with vesicles inside the dendrites and axons with myelin sheaths and unaltered organelles were seen. On the other hand, spleen cells appeared damaged in TTX-treated mice, mitochondria were mostly dilated with disintegration of cristae and matrix, cytoplasm had granular appearance due to the presence of abundant ribosomes (Figure 5d).

Ultrastructural cell damage is clearly remarkable in most organs related to the gastrointestinal tract, only stomach cells were not altered by TTX treatment (Figure 6b). TTX induced moderate liver damage, hepatocytes (Figure 6c,d) presented normal glycogen content and well developed rough endoplasmic reticulum (rer) surrounding the abundant mitochondria, which maintain their cristae well defined. However, lipid droplets increased their presence in hepatocyte cytoplasm of TTX-treated mice (Figure 6d). Furthermore, swelling of the rer cisternae was observed in enterocytes of small (Figure 7b,c) and large intestine (Figure 7e,f). Mitochondria in small and large intestine maintained their characteristic morphology but electron-dense granules were present inside the matrix. Nevertheless, microvilli density and morphology were not affected, and tight junctions preserved their structure intact. Nuclei and cellular membranes were not altered in any of the studied organs.

## 3. Discussion

Relevance of the public health risk associated to the presence of TTX in European seafood has led to numerous investigations [30]. The scientific opinion of the EFSA Panel on Contaminants in the Food Chain indicated the acute reference dose (ARfD) of 0.25 µg/kg to TTX [29] based on a recent study where the oral LD50 for TTX was established as 232 µg/kg bw and the oral NOAEL as 75 µg/kg bw [27]. However, EFSA pointed the need for more data on the oral toxicity of TTX [29]. Therefore, determination of the acute effects derived from ingestion of TTX represents an important step for the protection of the consumers.

Pufferfish containing TTX have caused serious seafood poisoning, especially in Japan where two to three people die annually since there is no specific antidote [16]. The TTX geographic expansion to Europe and diversification of TTX-bearing organisms (gastropods, crustaceans, and molluscs) [3] has made this toxin recognized as an emerging risk by European regulators [29]. In any case, the symptoms of poisoning due to the consumption of TTX vectors depend on the amount of toxin ingested [31]. Normally, onset of clinical signs begin very fast, within 30 min to 3 hours after consumption of TTX vectors, indicating an acute intoxication [6,31,32].

Since TTX toxicity data from human cases are rarely available, toxicokinetics and acute effects have been investigated in animal models [27,28,33]. Recently, some studies demonstrated that in rats TTX was rapidly absorbed, found in plasma 10 min after intramuscular treatment, and distributed mainly to the stomach, kidney, and intestines [28]. Furthermore, TTX was detected in blood samples from mice 2 h after oral gavage administration [27]. However, information about organ injuries due to TTX ingestion is actually scarce. 

With the goal to investigate the mechanism involved in TTX acute oral toxicity at the organ level, we studied the effects in mice. In our experiments, necropsy showed only macroscopic alterations in organs of the digestive tract such as stomach or intestine. Accordingly, TTX accumulation was previously detected in the intestine hours after the treatment [28]. Therefore, gastrointestinal alterations could be related to some symptoms developed in severe TTX poisoning (grade 1 when classified by a scale created by Fukuda and Tani in 1941) [25,32,34]. In spite of the evident macroscopic alteration found in stomach of animals treated with TTX, the TEM study did not reveal any further change in the cells of this organ, which is consistent with results previously found after chronic treatment with low TTX doses [35]. 

Conversely, necropsy did not indicate any alteration of spleen, but electron microscopy analysis revealed its cells sensitivity to TTX, evidenced by the presence of dilated mitochondria. Massive entry of water inside mitochondria can induce its swelling. These organelles supply energy to the cells and participate in cell signaling pathways, so any affectation of mitochondria structure and function could disturb the cell processes. Mitochondrial dysfunction can also contribute to trigger mitophagy [36] and even cell death by apoptosis or necrosis in severe cases, caused by an energy decrease or by an excessive production of reactive oxygen species (ROS) [37]. The opening of mitochondrial permeability transition pores (MPTPs) has also been documented as a previous event in the progression to apoptotic or necrotic cell death [38,39]. Alterations in cristae and inner membrane rupture are also indicatives of these types of cell death even though in spleen cells autophagosomes and fracture of mitochondrial membranes were not seen.

In small and large intestine, however, only electron-dense granules inside the matrix of mitochondria were detected. The presence of these structures has been related to ion accumulations. It was previously reported for other marine toxins that mitochondria swelling can be induced by water influx triggered by a previous huge overload of ions [40]. However, in intestinal cells from TTX-treated mice mitochondria size did not increase. 

Mice treated with TTX showed enterocytes of the small and large intestines with swollen rer cisternae. Rer is involved in protein synthesis. Retention or liberation of these proteins is closely related to dilation of the organelle, either by synthesis in excess or occurrence of some aberration in the production or transport mechanism [41]. Modifications in water and ion contents also may cause swelling of the organelle. In injured cells, the rer dilation is one of the earliest alterations observed, nevertheless these changes can be reversed if the stress inducer disappears [42], Alterations in mitochondria and rer were previously reported in the small and large intestines of mice orally treated with the marine toxin dinophysistoxin-2 (DTX2), as well as the possible inhibition of gastric emptying reflected by stomach dilation [43]. However, these toxins have a different mechanism of action. TTX is a neurotoxin and DTX2 belongs to the group of diarrheic shellfish toxins, although okadaic acid (OA) has been related to a neuronal action [44]. On the other hand, an increase of the number of lipid droplets was noted in hepatocytes that have been also related to cell death [45]. The absence of heart alterations agree with the lack of affinity for Nav1.5 sodium channels mainly expressed in the heart [24,46]. Nevertheless, the lack of TTX effect on heart and kidney in our acute study contrasts with the damage described in chronic treatments with repeated exposure of mice to TTX for 28 days. Therefore, only the continuous exposure to this toxin triggers renal damage associated with an increased permeability of the glomerular filtration barrier, together with a disintegration of heart myofibrils [35]. All these data could provide important hints about the acute adverse effects due to TTX ingestion and raise a serious concern for people’s health. 

## 4. Conclusions

The obtained results demonstrate that oral TTX induced macroscopic changes in stomach and intestine, and ultrastructural effects in spleen, hepatocytes, small and large intestines 2 h after exposure in mice. These findings suggest that gastrointestinal effects should be considered when evaluating human TTX poisoning.

## 5. Materials and Methods 

### 5.1. Materials

Tetrodotoxin (TTX) was supplied by CIFGA S.A. (Lugo, Spain). Serum 5% glucose and saline solution (0.9% NaCl) used in mice were from B. Braun VetCare S.A. (Barcelona, Spain) and Grifols (Barcelona, Spain), respectively. Sodium cacodylate trihydrate and 2.5% glutaraldehyde were purchased from Sigma-Aldrich Quimica S.A. (Madrid, Spain) as well as the rest of chemicals, which are analytical grade. Distilled water was purified using a water purification system (Milli-Q, Merck Millipore, Madrid, Spain). Metabolic cages for single mouse were from Tecniplast (Buguggiate, Italy).

### 5.2. Animals and Experimental Conditions

In vivo studies were performed following an Up and Down Procedure starting at 1000 µg/kg bw as previously described [27]. Briefly, Swiss female mice weighing 18–21 g were fasted overnight, with 5% glucose serum ad libitum, previously to oral administration of the toxin. A single oral dose of different TTX treatments (25, 75, 125, 250, 500, and 1000 µg/kg bw) diluted in 0.9% saline solution was administered by oral gavage (10 mL/kg) the next morning. Then mice were placed individually in metabolic cages for the following 2 h with free access to chow and water. When the time established for the experiment duration (2h after toxin administration) elapsed, the mice that survived were euthanized by CO_2_ inhalation.

All animal procedures described in the manuscript were carried out in conformity to European legislation (EU directive 2010/63/EU) and Spanish legislation (Real decreto 53/2013, Decreto 296/2008) and to the principles approved by the Institutional Animal Care Committee of the Universidad de Santiago de Compostela under the procedure Code: 01/17/LU-002 (approved on 22 September 2017).

### 5.3. Necropsy

All animals in the study were subjected to a full necropsy. Samples of several organs (heart, lung, brain, spleen, liver, kidney, stomach, small and large intestines) were collected after death or after euthanasia in mice that survived the whole experiment. During sample collection, macroscopic alterations were evaluated.

### 5.4. Sample Preparation for Transmission Electron Microscopy (TEM)

Mice organs were prepared for TEM immediately after extraction. One mm^3^ fractions from each organ were immersed in 2.5% glutaraldehyde in 0.1 M sodium cacodylate trihydrate buffer for 4 h at 4 °C for fixation. After that, samples were embedded in 0.1 M sodium cacodylate trihydrate buffer at the same temperature. Postfixation was performed by immersion in 1% osmium tetroxide (OsO_4_) in 0.1 M sodium cacodylate trihydrate buffer was performed for 60 min. Then, samples were rinsed again and fixed tissues were dehydrated in graded ethanol solutions, including one bath with 70% ethanol and 0.5% uranyl acetate. Finally, tissues were rinsed in propylene oxide, and embedded in Epon 812 (Momentive Speciality Chemicals Inc., Houston, TX, USA). A Leica Ultracut UCT ultramicrotome (Leica Microsystems GmbH, Wetzlar, Germany) was used to cut ultrathin sections of tissue samples, (1 mm^2^), and they were counterstained with uranyl acetate and lead citrate.

Ultrastructural analysis of 1 mm^2^ sections was performed with a JEOL JEM-1011 Transmission Electron Microscope (Jeol Ltd, Tokyo, Japan).

## Figures and Tables

**Table 1:** Mortality and symptoms observed after TTX administration indicated as a percentage of affected mice versus total treated animals.

TTX Dose (µg/kg bw)	Mortality (%)	Symptoms (%)				
		Seizures	Squint Eyes	Circling Behavior	Numbness	Apathy
25	No mortality	0	0	0	0	0
75	No mortality	0	0	0	0	0
125	No mortality	0	0	0	0	100
250	57%	28.6	0	14.3	28.6	100
500	80%	40	20	40	40	100
1000	100%	100	0	0	100	100
